# Graphene Oxide-Based Nanostructured DNA Sensor

**DOI:** 10.3390/bios9020074

**Published:** 2019-05-30

**Authors:** Aditya Balaji, Songlin Yang, Jeslyn Wang, Jin Zhang

**Affiliations:** 1 Department of Biomedical Engineering, University of Western Ontario, London, ON N6A 5B9, Canada; abalaji2@uwo.ca; 2 Department of Chemical and Biochemical Engineering, University of Western Ontario, London, ON N6A 5B9, Canada; syang368@uwo.ca (S.Y.); Jeslyn.Wang2019@gmail.com (J.W.)

**Keywords:** DNA sensor, fluorescent magnetic core-shell nanoparticles, graphene oxide, FRET quenching mechanism

## Abstract

Quick detection of DNA sequence is vital for many fields, especially, early-stage diagnosis. Here, we develop a graphene oxide-based fluorescence quenching sensor to quickly and accurately detect small amounts of a single strand of DNA. In this paper, fluorescent magnetic nanoparticles (FMNPs) modified with target DNA sequence (DNA-t) were bound onto the modified graphene oxide acting as the fluorescence quenching element. FMNPs are made of iron oxide (Fe_3_O_4_) core and fluorescent silica (SiO_2_) shell. The average particle size of FMNPs was 74 ± 6 nm and the average thickness of the silica shell, estimated from TEM results, was 30 ± 4 nm. The photoluminescence and magnetic properties of FMNPs have been investigated. Target oligonucleotide (DNA-t) was conjugated onto FMNPs through glutaraldehyde crosslinking. Meanwhile, graphene oxide (GO) nanosheets were produced by a modified Hummers method. A complementary oligonucleotide (DNA-c) was designed to interact with GO. In the presence of GO-modified with DNA-c, the fluorescence intensity of FMNPs modified with DNA-t was quenched through a FRET quenching mechanism. Our study indicates that FMNPs can not only act as a FRET donor, but also enhance the sensor accuracy by magnetically separating the sensing system from free DNA and non-hybridized GO. Results indicate that this sensing system is ideal to detect small amounts of DNA-t with limitation detection at 0.12 µM.

## 1. Introduction

Since the well-known Human Genome Project unveiled the human sequence in 2000, we have been aware that almost all diseases are related to genetic disorder [1,2,3,4]. Some genetic changes are inherited, while some are acquired during person’s life because of various factors. Detection of the mutation of gene or DNA sequences is important in order to have the early-stage diagnosis and personalized therapeutics [5,6].

The main methods for detecting DNA sequences include polymerase chain reaction (PCR), radioisotopes, intercalating dyes exposed with UV light, and silver staining process. Among them, PCR is a powerful tool to analyze samples of DNA sequences apart from amplifying minute amounts of nucleic acids. However, these methods are expensive and need a long time for the detection to occur [7,8,9,10]. With the development of fluorescence nanomaterials, e.g., quantum dots, dye-loaded silica nanoparticles, etc., fluorescent detection has become an attractive method for its low cost, high sensitivity, and low background noise. In addition, the special properties of nanomaterials, e.g., high percentage of surface atom number, large surface area to volume ratio, make them an ideal transducer to bind a large amount of oligonucleotides to quickly convert the DNA replication to a fluorescence signal [11].

Fluorescence resonance energy transfer (FRET) is a distance dependent, nonradioactive process. This process normally composes the excited state fluorophore (donor) and a nearby ground state fluorophore (acceptor). Under external light excitation, emission of the donor will excite the acceptor through the fluorescence energy transfer. The efficiency *E*, refers to the quantum yield of the energy transfer transition, which depends on the distance between the donor and the acceptor, <10 nm, as shown the equation below [12];

$$E=\frac{R_{0}^{6}}{R_{0}^{6}+r^{6}},$$

where *r* is the actual distance between donor and acceptor, and *R*_0_ is the Förster distance at which the energy transfer efficiency is 50% [12,13,14,15].

For the FRET quenching mechanism, the acceptor should be able to have an absorbance at the wavelength that overlaps with the emission of the donor. Recently, the FRET quenching mechanism has been applied to study protein–protein interaction and bioimaging [16,17]. 

Graphene oxide (GO) is a two-dimensional (2D) crystal structure made of carbon, and arranged in a hexagonal pattern with various oxygen functional groups on the surface, e.g., carboxyl, hydroxyl and epoxy groups [18]. GO has demonstrated potential in bio-sensing applications as it is considered as a stronger quenching element due to the sp^2^ hybridization, π–π* and/or n–π* orbital interactions, and its versatile bioconjugation chemistries [19,20]. Previous studies indicate that the distance between the GO and fluorophores is critical in the design of GO quenching-based sensors [21].

On the other hand, multifunctional nanoparticles made of a magnetic core and fluorescent-shell (FMNPs) have been used as a donor because of their good photostability; furthermore, the separation of biomolecules can be easily realized through binding to FMNPs [22,23]. Thus, the magnetic properties of FMNPs can be applied separately from free DNA sequences, and, therefore, can accurately determine the amount of DNA sequences conjugated on FMNPs.

**Figure 1:**
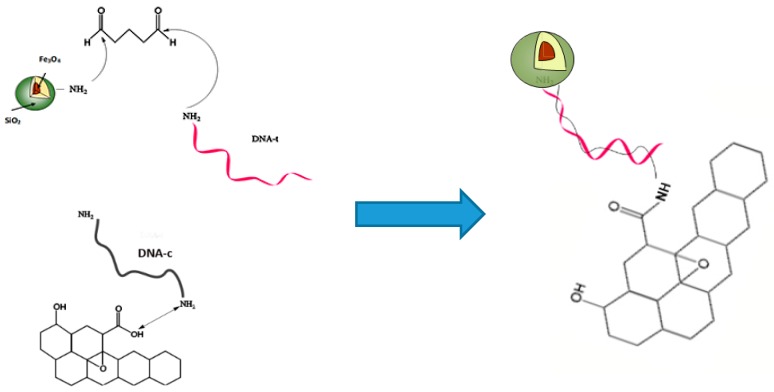
The sensor design for detecting target DNA by using the fluorescence resonance energy transfer (FRET) quenching mechanism.

In this paper, target DNA sequences (DNA-t) are conjugated onto FMNPs that can not only provide fluorescence emissions, but also have the capability to remove the extra DNA-t, therefore enhancing sensing accuracy. In addition, the major drawbacks of using only a magnetic nanostructure may be related to the aggregation and the oxidation exposed with air, resulting in the loss of magnetism and dispersibility. Silica coating can prevent the aggregation and oxidation of the magnetic core. Figure 1 shows the covalent interaction between MFNPs and DNA-t with 5′-end modification through glutaraldehyde crosslinking. The complementary oligonucleotide, i.e., capture DNA (DNA-c), is modified with the amine group where it can interact with the functional group, −COOH, on GO. When DNA hybridization occurs, the fluorescence intensity of the donor, e.g., FMNPs, will be supressed by the quencher, GO. The correlation of fluorescence quenching and the level of DNA hybridization can determine how much DNA-t is in a system. 

## 2. Materials and Methods

### 2.1. Synthesis of Fluorescent Magnetic Core-Shell Nanoparticles Modified with Target Oligonucleotide

FMNPs made of the magnetic core, Fe_3_O_4_, and rhodamine B isothiocyanate (RITC)-loaded silica shell, were produced by thermal-decomposition following an oil-in-water micro-emulsion process [23]. Briefly, 10.8 g of iron chloride (FeCl_3_·6H_2_O, 40 mmol, Aldrich, 98%) and 36.5 g of sodium oleate (120 mmol, TCI, 95%) were dissolved in 300 mL of a mixture of ethanol, distilled water, and hexane. The solution was increased to 70 °C using a thermostat and kept for four hours. When the reaction was completed, the upper organic layer, which contained the iron-oleate complex was washed three times. The iron-oleate complex was further dissolved in oleic acid and 1-octadecene. The temperature of the mixed solution was increased to 320 °C with a kinetic rate of about 3 °C min^−1^. Once the temperature was saturated at the desired temperature, the solution was kept for 30 min. A severe reaction occurred where the transparent solution became brownish black. The resulting solution containing Fe_3_O_4_ nanocrystals was then cooled to room temperature, and 167 mL of ethanol was added to precipitate the nanocrystals. The nanocrystals were separated by centrifugation as a last step process.

Furthermore, 10 mg of rhodamine B isothiocyanate (RITC) was reacted with 44 µL of 3-aminopropyltriethoxysilane (APTES) (molar ratio of RITC: APTES = 1:10) in 0.75 mL of ethanol under dark conditions for 2 days. Following this, 0.5 mL of Fe_3_O_4_ nanocrystals in chloroform was mixed with 5 mL of aqueous cethyltrimethylammonium bromide (CTAB) solution. After vigorous shaking, the formation of an oil-in-water micro-emulsion resulted in a turbid brown solution. The mixture was heated to 60 °C and stabilized at this temperature for 10 min to evaporate the chloroform, resulting in a transparent black Fe_3_O_4_/CTAB solution. Next, 45 mL of water and 0.3 mL of 2 M NaOH was added where the mixture was heated to 70 °C under stirring. Then, 0.5 mL of tetraethylorthosilicate (TEOS), 50 μL of RITC-APTES solution, and 3 mL of ethylacetate was added to the reaction solution in sequence. After 10 min, 50 μL of APTES was added and the solution and was stirred for 3 h. The synthesized FMNPs were washed with a series of steps to remove the unreacted species and extract CTAB.

The target oligonucleotide (DNA-t), 5′ CTT TTG TTC 3′, in a stock solution was prepared by dissolving in 1.9 mL of Tris-HCl buffer at pH 7.4. The amino group modified 5′ end DNA was achieved by using the carbodiimide crosslinker, N′-(3-dimethylaminopropyl)-N-ethylcarbodiimide (EDC) and imidazole [24]. First, 6.52 μmol of EDC was mixed with the prepared oligonucleotide. After introducing the 0.25 M ethylenediamine and 0.1 M imidazole solution, the mixture was incubated at 37 °C for 2 h. Non-reacted EDC and its by-products and imidazole were removed by dialysis. The amino group modified 5′ end DNA was transferred into a glass vial and dispersed in 3 mL of HEPES Buffer (20 mM HEPES, 150 mM NaCl, pH 7.4) to obtain a final concentration of 0.1 µM. Following the addition of 50 µL glutaraldehyde, the 5′ end modified DNA was reacted with amino modified FMNPs overnight and incubated at 40 °C [25].

### 2.2. Synthesis of Graphene Oxide Nanosheets Modified with Capture Oligonucleotide (DNA-c)

A modified Hummer’s approach was applied in this study to produce graphene oxide nanosheets (GO) [26]. While stirring in an ice bath, 1 g of graphite flakes was added to 50 mL concentrated sulfuric acid. Following this, 3 g of potassium permanganate was slowly added by maintaining a temperature of under 10 °C to obtain graphite oxide. The suspension was stirred at room temperature for 25 min followed by 5-min sonication in an ultrasonic bath where this process was conducted 12 times. The reaction was quenched by the addition of 200 mL distilled water and an extra 2-h ultrasonic treatment was carried out, which produced graphene oxide. The pH value was adjusted to 6 by the addition of NaOH, and the suspension was further sonicated for 1 h. 

Complementary oligonucleotide, DNA-c, with the sequence of 5′ GAA AAC AAG 3′ was modified with amino group at 5′ end by using the same technique used for DNA-t [24]. The strand was kept in the Tris-HCl buffer at pH~6. The GO was prepared at around pH~6 and so the carboxylic acid groups were not deprotonated and bonded to DNA-c through peptide bonding by using EDC associated N-hydroxysuccinimide (NHS) [24,27,28]. Briefly, 2 mg EDC and 1 mg NHS were added into GO (1 mg/mL) solution with a 2 h incubation at 37 °C to activate carboxyl groups on the surface of GO. Different amounts of GO were used to react with 100 µL of 10 µM DNA-c to determine the optimal ratio of GO to DNA-c. The solutions were incubated for 2 h at 37 °C. The DNA-c binding GO was obtained after dialysis and kept under 4 °C. The standard curve of DNA concentration was measured by using UV-Vis spectrometer (see the supplementary materials). The optimal ratio of FMNPs to DNA-t, and the optimal ratio of GO to DNA-c were determined. 

### 2.3. Characterization Methods

Microstructures of nanomaterials were studied by using Phillips CM 10 TEM. Fourier Transform Infrared Spectroscopy (FTIR, Bruker Vector 22 FT-IR spectrometer, Milton, ON, Canada) was carried out to investigate the functional groups. The scan range for the FT-IR was 600 to 4000 cm^−1^ and the resolution was set at 1 cm^−1^. The absorbance of DNA molecules with different concentrations was studied by using an Ultraviolet-visible spectrophotometry (UV-Vis, Agilent Cary 60 UV-Vis Spectrophotometer, Santa Clara, CA, USA). The magnetic properties Fe_3_O_4_ nanoparticles and core-shell nanoparticles (FMNPs) were studied by using the vibrating sample magnetometer (VSM, LakeShore 7407 vibrating sample magnetometer, Westerville, OH, USA). The photoluminescence properties of FMNPs were studied by using QuantaMaster™ 40 Spectrofluorometer (Horiba Canada–Photon Technology International Inc., London, ON, Canada). The DNA hybridization was detected by using the Spectrofluorometer. 

### 2.4. Detection of Hybridization of DNA-t and DNA-c 

DNA hybridization was investigated under simulated physiological conditions (pH 7.4). All fluorescence measurements were performed under an excitation wavelength at 520 nm. The dried DNA-c conjugated GO powder was suspended in PBS to obtain different concentrations. 100 μL of GO-DNA-c with different concentrations was mixed with 1 mL FMNPs, binding with a 20 μM DNA-t (FMNPs-DNA-t) solution. The mixture was incubated at 37 °C for 20 min in a dark environment. After incubation, the solution was treated with ultrasonic following the magnetic confinement to remove the extra DNA and GO. The fluorescence signal of samples in 1 mL was measured to evaluate the level of hybridization and determine the amount of target DNA sequence. 

## 3. Results and Discussion

**Figure 2:**
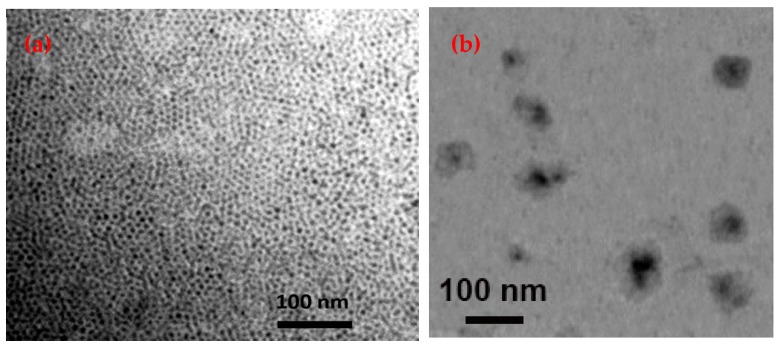
TEM micrographs of (**a**) Fe_3_O_4_, (**b**) Fe_3_O_4_ nanoparticles and core-shell nanoparticles (FMNPs).

The TEM micrograph of monodispersed Fe_3_O_4_ NPs shows the nanoparticles with diameter of 12 ± 3 nm (Figure 2). Furthermore, the core-shell FMNPs had an average particles size of 74 ± 6 nm and the average thickness of the silica shell, estimated from TEM results, was 30 ± 4 nm.

**Figure 3:**
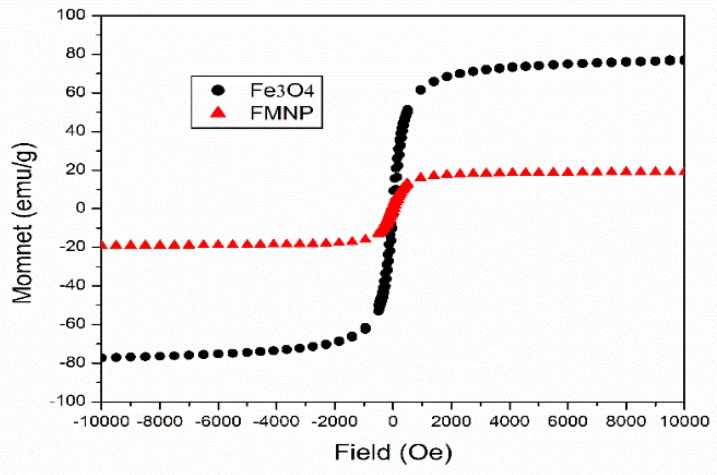
Hysteresis loops of Fe_3_O_4_ NPs and core-shell FMNPs.

The magnetic FMNPs used here were modified with DNA-t to act as a donor, but also were used to increase sensing accuracy by separating the non-reacted DNA-t from the system by an external magnetic field. Figure 3 shows the magnetic properties of Fe_3_O_4_ with magnetic moment over 80 emu/g at a field of 10 KOe. The nanoparticles showed very low coercivity, which indicated their superparamagnetic properties. When Fe_3_O_4_ nanoparticles were coated with silica shall, the magnetic moment decreased dramatically to 20 emu/g because of the large amount of silica.

**Figure 4:**
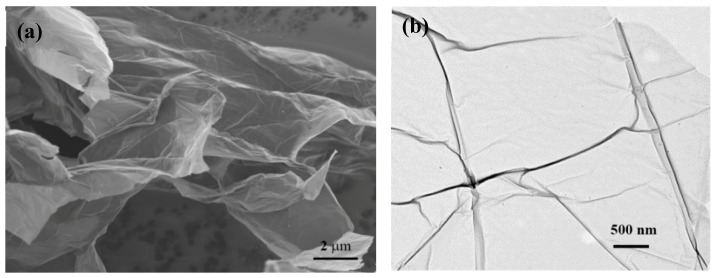
Electron micrographs of graphene oxide: (**a**) SEM micrograph of graphene oxide nanosheet. (**b**) TEM micrograph of graphene oxide nanosheet.

On the other hand, the GO made by the solution method was investigated. Figure 4 shows the SEM micrograph (Figure 4a) and TEM micrograph.

**Figure 5:**
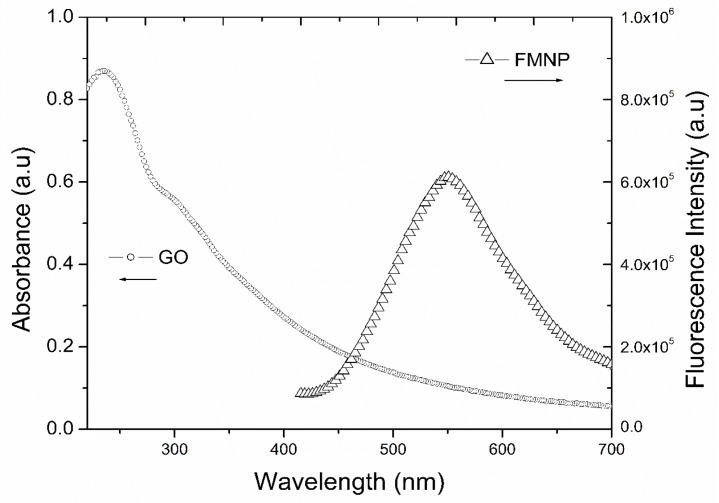
Photoluminescence of FMNPs with the excitation wavelength.

GO was used as the fluorescent quencher. Figure 5 shows that GO has a very broad absorbance in the range of 200 nm to 700 nm. The maximum absorbance, centering at 250 nm due to the π-plasmon of the sp^2^ carbon structure; and the absorbance around 300 nm is attributed to n–π* transition [21]. It is also noted that the broad absorbance of GO covers the major emission range of FMNPs with the maximum fluorescence intensity centering at 575 nm.

**Figure 6:**
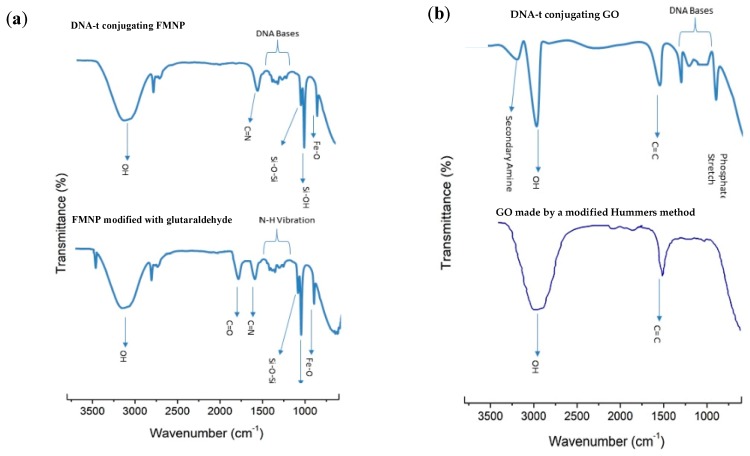
FTIR spectra (**a**) FMNPs conjugating with glutaraldehyde, and DNA-t modified. FMNPs, (**b**) graphene oxide (GO) nanosheet, and DNA-c-modified with GO.

In addition, the FTIR spectra of glutaraldehyde-modified FMNPs and FMNPs modified with DNA-t are displayed in Figure 6a. The asymmetric stretch of Si–O–Si (1000 cm^−1^), C=N stretch (1600 cm^−1^), C=O stretch (1800 cm^−1^) were observed, as shown in Figure 6a and Figure 6b. The peak of around 3400 cm^−1^ arose due to the compound being suspended in water, which corresponds to the –OH group. The FTIR spectrum of FMNPs modified with DNA-t shows the Fe–O stretch at 700 cm^−1^, and an asymmetric stretch of Si–O–Si (1000 cm^−1^). In Figure 6a, the small signal peaks in the range of 1000 cm^−1^ to 1400 cm^−1^ are attributed to DNA structure. The C=N stretch is observed at 1600 cm^−1^.

The FTIR spectrum of GO in Figure 6b shows the typical −OH stretch at 3000 cm^−1^, and C=C stretch at 1500 cm^−1^. In addition, peaks at 2362 cm^−1^, 1699 cm^−1^ and 1221 cm^−1^ are attributed to the stretching vibrations of C–OH, C=O, and C–O, respectively. Figure 6b shows the FTIR spectrum of the sample of DNA-c conjugating on GO. The vibration of phosphate backbone can be observed around 800 cm^−1^; the peaks in the range of 930 cm^−1^ to 1218 cm^−1^ are attributed to DNA bases; the stretch of C=O is observed around 1361 cm^−1^; the peak at 1562 cm^−1^ is attributed to the stretch of C=N; The stretch of −OH is around 3038 cm^−1^, and the peak of 3267 cm^−1^ is attributed to the stretch of secondary imine.

The standard curve of the absorbance of the DNA sequence with the concentration was obtained and is shown in Figure S1 (see the supplementary materials). In aqueous solutions, the ratio of FMNPs to DNA-t was estimated at 1 mg/mL to 4 µM; while the ratio of GO to DNA-c was maintained with 1 mg/mL to 10 µM. 

**Figure 7:**
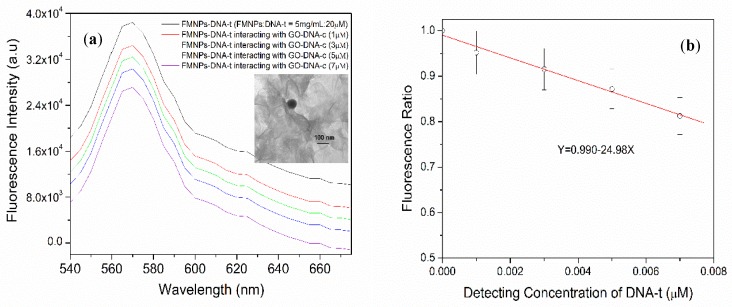
(**a**) The fluorescence quenching performance when the DNA-t modified FMNPs interacting with different concentrations of DNA-c modified GO nanosheet. The small inset is the TEM of the FMNP-DNA-t covalently binding to GO-DNA-c. (**b**) Fluorescence ratio vs. detecting concertation of DNA-t by the solution sensing system.

The interaction between DNA-t bound onto FMNPs, and GO with DNA-c were studied. In an aqueous solution, 100 µL of DNA-c conjugating GO was mixed with 1 mL FMNP-DNA-t (20 µM) through DNA hybridization. The fluorescence intensity of the MFNPs decreased when increasing the concentration of DNA-c binding with GO. Due to the hybridization of DNA-t and DNA-c occurs at the ratio of 1:1, we are able to determine the decrease of fluorescence intensity as a function of the concentration of DNA-t. Figure 7a shows the photoluminescence of DNA-t binding FMNPs (FMNPs-DNA-t) which react with the different concentrations of DNA-c dinging GO (GO-DNA-c). The fluorescence intensity of FMNPs-DNA-t decreases when GO-DNA-c is introduced, and the concentration of introduced DNA-c increases from 1 µM to 7 µM. The small inset is the FMNPs binding on GO through DNA hybridization. It is noted that non-reacted GO was removed through magnetic confinement. Figure 7b is the fluorescence ratio as a function of the concentration of the detected DNA-t. Here, the fluorescence ratio refers the normalized fluorescence intensity, i.e., dividing the fluorescence intensity of FMNPs-DNA-t reacting with GO-DNA-c by that of the FMNPs-DNA-t. The regression equation is expressed as y = 0.99 − 24.98x with a correlation coefficient R^2^ of 0.98463, where y is the fluorescence intensity and x is the corresponding concentration of DNA-t, which can be hybridized with DNA-c on GO. The detection limit, based on 3 σ/slope (where σ was the standard deviation of the low concertation) [29], is 0.12 µM.

## 4. Conclusions

In summary, fluorescent magnetic nanoparticles incorporated with graphene oxide is a suitable FRET pair for DNA detection. DNA-t and DNA-c were conjugated onto the nanostructured donor and acceptor, respectively. Core-shell FMNPs were produced by a thermal-decomposition process following silica coating. The average particle size of FMNPs is 74 ± 6 nm and the average thickness of the silica shell, estimated from TEM results, is 30 ± 4 nm. The magnetic properties of FMNPs have been investigated, and show 20 emu/g magnetic saturation under 10 KOe. Our study demonstrates that FMNPs are suitable FRET donors and can provide stable fluorescent intensity with emissions at 575 nm, and can be used to magnetically separate extra DNA and GO from the system in order to enhance sensing accuracy. When DNA hybridization occurs, the GO acts as the quenching element due to its typical sp^2^ hybridization and π–π*/n–π* orbital interactions. Therefore, the fluorescence intensity of FMNPs decreases through the FRET quenching mechanism. The results indicate that the limit of detection is as low as 0.12 µM, and optimal detection range in this study is from 0 to 10 µM of DNA-t in this system.

## Supplementary Materials

The following are available online at https://www.mdpi.com/2079-6374/9/2/74/s1, Figure S1: UV absorbance of DNA with different concentrations in aqueous solution.
