# Supplementary material for: Graphene Oxide-Based Nanostructured DNA Sensor
Source: Biosensors (Basel). 2019 May 30;9(2):74. doi: 10.3390/bios9020074 (PMC6627418; doi:10.3390/bios9020074)
Supplement: Supplementary file 1 [file biosensors-09-00074-s001.pdf]

Supplementary Materials

Graphene Oxide-Based Nanostructured DNA Sensor

Aditya Balaji [1], Songlin Yang [2], Jeslyn Wang [2] and Jin Zhang [1,2,*]

[1] Department of Biomedical Engineering, University of Western Ontario, London, ON N6A 5B9, Canada; abalaji2@uwo.ca

[2] Department of Chemical and Biochemical Engineering, University of Western Ontario, London, ON N6A 5B9, Canada; syang368@uwo.ca (S.Y.); Jeslyn.Wang2019@gmail.com (J.W.)

* Correspondence: jzhang@eng.uwo.ca; Tel.: +1-519-661-2111

The Standard curve of DNA concentration was measured by using UV-Vis spectrometer

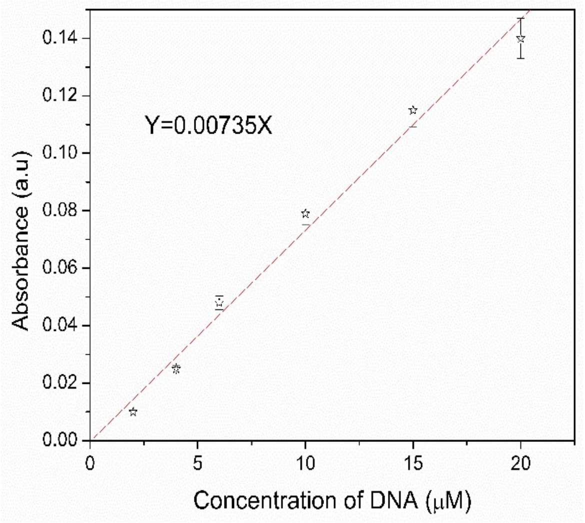

Figure S1. UV absorbance of DNA with different concentrations in aqueous solution.
